# Chronic unpredictable mild stress-induced mouse ovarian insufficiency by interrupting lipid homeostasis in the ovary

**DOI:** 10.3389/fcell.2022.933674

**Published:** 2022-09-08

**Authors:** Yongjie Xiang, Lin Jiang, Junjie Gou, Yibo Sun, Dongyu Zhang, Xigeng Xin, Zhenhua Song, Jiaojiao Huang

**Affiliations:** ^1^ College of Animal Science and Technology, Qingdao Agricultural University, Qingdao, China; ^2^ Center for Reproductive Medicine, Qingdao Women and Children’s Hospital, Qingdao University, Qingdao, China; ^3^ Department of Neurosurgery, Center Hospital of Yantai, Yantai, China; ^4^ School of Pharmacy, Qingdao University, Qingdao, China

**Keywords:** CUMS, ovarian insufficiency, E2, lipid homeostasis, apoptosis

## Abstract

Ovarian insufficiency results from a number of disorders, and a certain causal relationship between psychological stress and ovarian insufficiency has been reported, but the underlying mechanism remains unclear. In our study, C57BL/6J female mice were subjected to chronic unpredictable mild stress (CUMS), and depression-like mice were selected and identified according to the behavioral tests. The defective ovarian follicle development, low 17 β-estradiol (E_2_), and anti-Mullerian hormone (AMH) levels, which were consistent with the clinical characteristics of ovarian insufficiency, indicated that depression-like mice may be used to assess the effects of psychological stress on female reproductive function. To investigate a possible mechanism, lipid homeostasis of the ovary was detected by liquid chromatography tandem mass spectrometry (LC-MS/MS) analysis, and the decreased abundance of cholesteryl ester (CE 24:4) was supported to be associated with the downregulated E_2_. Moreover, granulosa cells did undergo more apoptosis in response to psychological stress, which was caused by downregulated *Bcl2* and *Bcl2/Bax* in granulosa cells. Additionally, the disorder of cell death and growth-related pathways in depression-like mouse ovaries was confirmed by RNA-seq analysis. Taken together, this study will provide a better understanding of the female reproductive problem under psychological stress.

## Introduction

Psychological stress in humans often refers to insufferable “emotional experiences” and is accompanied by biochemical, physiological, and behavioral changes that might have a major influence on mood, behavior, and health (Schneiderman et al., 2005). Studies have revealed that depression develops in roughly 20–25 percent of people who have experienced acute psychological stress (Wulsin et al., 2022). Additionally, an underlying connection between psychological stress and physical illness has been reported, including human immunodeficiency virus (HIV), cardiovascular disease (CVD), acquired immunodeficiency syndrome (AIDS), and cancer (Cohen et al., 2007). At present, accumulating evidence has demonstrated that psychological stress might affect female reproduction. Psychological stress has been linked to oocyte development, oocyte fertilization, embryonic development, pregnancy, live birth delivery, birth weight, and multiple gestations in women (Klonoff-Cohen et al., 2001; Neggers et al., 2006; Williams et al., 2007; Stickel et al., 2019; Zhai et al., 2020).

Ovarian insufficiency can be caused by a primary disorder in the ovary, or it can occur as a result of secondary causes. Ovarian insufficiency is considered primary if the ovary fails to function normally in response to appropriate gonadotropin stimulation provided by the hypothalamus and pituitary. In secondary ovarian insufficiency, the ovaries are normal, but there is a problem getting hormone signals to them from the brain. Recent studies have shown that the occurrence of ovarian insufficiency, which is an ovarian abnormality marked by premature ovarian follicle depletion and significantly decreased 17-estradiol (E_2_) levels before the age of 40 years, is reported to be closely linked to anxiety, depression, and other negative emotional states in women (Kaplan and Manuck, 2004; Pal et al., 2010; Allshouse et al., 2015). Approximately, 43 percent of ovarian insufficiency patients have a history of depression, and roughly 26 percent of those patients have had depression within 5 years of being diagnosed with ovarian insufficiency (Allshouse et al., 2015). The ovarian reserves of C57BL/6 mice (Gao et al., 2020) and SD rats (Fu et al., 2018; Fu et al., 2020) have been reduced by chronic unpredictable mild stress (CUMS) treatment, according to several animal studies. It was reported that the numbers of primordial follicles (*p* < 0.05) and preantral follicles (primary follicle, *p* < 0.01; secondary follicle, *p* < 0.05) were significantly lower in the chronic unpredictable stress group (Gao et al., 2020). Numerous researchers have found a link between female primary ovarian insufficiency (POI) and psychological stress, as previously mentioned (Xu et al., 2020; Li et al., 2021).

In terms of mechanism, when people experience psychological stress, the hypothalamic–pituitary–adrenal (HPA) axis is active, which results in high levels of corticotropin-releasing hormone (CRH) and glucocorticoids being released into the bloodstream (Stephens and Wand, 2012). It has been shown that glucocorticoids can impair the ovarian function by suppressing the synthesis and release of GnRH in the hypothalamus, which results in decreased LH and FSH production in the pituitary gland (Gore et al., 2006). Also, the suppression of BDNF-PI3K-Akt-mTOR signaling molecules might induce follicle development abnormalities under CUMS treatment (Li et al., 2021). In addition to this, glucocorticoids can interfere with the production of testosterone *via* modulating the glucocorticoid receptor (GR) or directly triggering apoptosis, resulting in a reduction in the female reproductive function (Grad and Picard, 2007). Moreover, it is reported that the administration of glucocorticoids stimulates the production of progestins through the accumulation of lipid, especially cholesterol esters (Towns et al., 1999). Cholesterol has an important influence on mammalian follicular development through regulating ovarian sex hormone synthesis as a key substrate. The knockout of cholesterol transport-related receptors (HDL and LDL) in the mammalian cells results in the serious reduction or absence of fertility, leading us to recognize the importance of cholesterol homeostasis in the ovary to female reproduction (Huang et al., 2019). Despite this, little is known about the impact of psychological stress on ovarian cholesterol metabolism and the complicated management of its homeostasis in the ovaries.

In our study, C57BL/6J female mice were subjected to chronic unpredictable mild stress, and depression-like mice were selected and identified according to the behavioral tests. Ovary morphology and plasma levels of reproductive hormone were then assessed in depression-like mice. The ovarian follicle deficiency and low estradiol levels, which were consistent with the clinical characteristics of ovarian insufficiency, indicated that depression-like mice may be utilized to investigate the impact of psychological stress on female reproductive function. To study a possible mechanism, we examined the overall composition of lipid classes and the total transcriptome in the mouse ovary. This study will provide a better understanding of the female reproductive problem under psychological stress.

## Materials and methods

### Mice

All of the experiments were performed using female mice with C57BL/6J strain, which were purchased from Beijing Vital River Laboratory Animal Technology Co., Ltd. (Beijing, China). Mice were fed in the cages (32 cm × 16 cm × 16 cm) under controlled environmental conditions. The experimental protocols were approved by the Animal Use and Care Committee of Qingdao Agricultural University.

### Depression-like mice induced by chronic unpredictable mild stress

A group of 35-day-old female mice were selected for the experiments. First, the mice were adapted for 1 week, and during this period, self-control data including Y-maze tests (YMTs), forced swimming tests (FSTs), and sucrose preference tests (SPTs) were determined. Then, mice were divided into the control and CUMS-treated groups. Following that, the mice in the CUMS-treated group were fed for 6 weeks under the CUMS environment, according to the previous description (Xu et al., 2016; Si et al., 2018; Sun et al., 2018). After the consequent CUMS treatment, the depression-like behaviors of mice were accounted for by measuring the indexes of YMTs, FSTs, and SPTs. Mice with significant decreases in the values of SPT and the YMT above 20% of their self-control values, as well as a significant increase in values of FST above 15% of their self-control values, were defined as CUMS-induced depression-like mice. Only the depression-like mice have been used in the following research studies.

### Hormone measurement

After CUMS treatment, the mice were anesthetized, and blood samples were collected in heparinized centrifuge tubes. The obtained serum samples were prepared and stored at −80°C until analysis. Serum levels of mouse E_2_ and AMH were specifically measured with the enzyme-linked immunosorbent assay (ELISA) kits (Lanpai Biotechnology Co., Ltd., Shanghai, China).

### Ovary tissue collection and histological analysis

Following euthanasia, the mouse ovaries were removed and dissected immediately. Then, after fixing in 4% paraformaldehyde (PFA) at 4°C overnight, the fixed ovaries were cut into sections (5 μm thickness) and mounted on glass slides, then dehydrated, and embedded in paraffin. Following deparaffinization, the slides were stained with hematoxylin and eosin (HE) for histological analysis of follicles.

### Quantification of mouse ovarian follicles

In order to count the number of follicles in an ovary, the paraffin-embedded ovaries were cut serially into sections of 5 μm thickness, and then, every fifth section was mounted on slides, according to the previous description (Tilly, 2003). Based on the well-accepted standards set by Pedersen and Peters, ovarian follicles at various developmental stages were counted in collected sections of one ovary (Pedersen and Peters, 1968). In order to avoid the repetitive counting of the same follicles, only follicles containing oocytes with a visible nucleolus were counted.

### Granulosa cell isolation

First, the mice were injected with PMSG (7 IU), and then, euthanasia was carried out after 48 h. After dissecting the ovary, the large antral follicles were punctured in an M2 medium, and GCs were extruded. The GCs were harvested and washed with PBS, then mixed with TRIzol, and stored at −80°C.

### Real-time quantitative PCR analysis

Total RNA was extracted from the ovary or GCs using TRIzol, according to the manufacturer’s instructions. cDNA was synthesized using the PrimeScript RT Reagent Kit (RR037A; TaKaRa Bio Inc., Shiga, Japan). Real-time PCR was performed using a Bio-Rad CFX96 Touch system. Amplification of mRNA was performed in a 20 μl reaction with 1 μl of sample cDNA, 0.5 μl of each primer (10 nmol·l^−1^) (Supplementary Table S1), 10 μl of 2×qPCR Mastermix (Green), and 8 μl of ddH_2_O. PCR cycles were run at 94°C for 2 min, followed by 45 cycles of denaturation for 15 s at 94°C, annealing and elongation for 30 s at 60°C, and melt curve 65–95°C increase of 0.5°C for 5 s. All values were normalized to GAPDH to balance potential irregularities in the RNA concentration. All experiment runs were repeated in three replications. The 2^−ΔΔCt^ method was used to calculate the fold changes in gene expression.

### TUNEL analysis

TUNEL assays were performed with the *In Situ* Cell Death Detection Kit (Roche Diagnostics, Basel, Switzerland, 11684795910), according to the manufacturer’s instructions. As described by Song et al. (2015), after heating at 60°C for 2 h, the slides of ovary sections were first washed in xylene, then rehydrated through a graded series of ethanol and double distilled water, followed by treatment with proteinase K for 15 min at room temperature, finally incubated with the TUNEL reaction mixture in a humidified atmosphere for 60 min at 37°C in the dark, and counterstained with Hoechst for 5 min. The sections were observed for fluorescein labeling under a fluorescence microscope (Nikon, Tokyo, Japan).

### Lipid extraction and mass spectrometric analyses

The frozen ovaries were thawed on ice, then grinded, and resuspended using 120 µl of precooled 50% methanol buffer. The metabolites were extracted from 20 µl of each sample. After vertexing for 1 min and incubating for 10 min at room temperature, the sample was stored at −20°C overnight. After centrifugation at 4,000 g for 20 min, the supernatant was transferred to 96-well plates. Additionally, a pooled quality control (QC) sample was generated by mixing 10 µl of each extraction combination together. A TripleTOF 5600 Plus high-resolution tandem mass spectrometer (SCIEX, Warrington, United Kingdom) was used to evaluate all of the samples, which could be analyzed in both the positive and negative ion modes. In this study, an ultra-performance liquid chromatography (UPLC) system was used to perform the chromatographic separation (SCIEX, United Kingdom). The acquired LC-MS data pretreatment was performed and analyzed by LC-Bio Technology Co., Ltd. (LC-Bio, Hangzhou, China).

### Ovary RNA-seq analysis

Sequencing library preparation and RNA-seq were conducted at LC-Bio Technology Co., Ltd. (LC-Bio, Hangzhou, China). RNA samples with high purity (OD 260/280 ≥ 2.0) and high integrity (RIN >7) were used for cDNA library construction. The cDNA library construction, mapping reads, and FPKM calculations were conducted according to a previous study (Shen et al., 2019). Differentially expressed genes defined by *p* < 0.05 and an absolute fold change >1.5 were used for analysis.

### Pathway and process enrichment analysis

For upregulated and downregulated gene list, pathway and process enrichment analysis has been separately carried out using the “Metascape” Gene Annotation and Analysis Resource (https://metascape.org/gp/index.html#/main/step1). In order to collect and group terms based on their membership similarities, terms with a *p*-value less than 0.01, a minimum count of 3, and an enrichment factor greater than 1.5 (the enrichment factor is the ratio between the observed counts and the counts expected by chance) are collected and grouped into clusters.

### Statistical analysis

All the experiments were repeated at least three times, and all the measured data were presented as the mean ± SD. Student’s *t*-test was adopted to analyze the comparison between control and depression-like mice with SPSS statistical software (IBM Co., NY). The differences between groups were considered statistically significant if *p* < 0.05.

## Results

### Depression-like mice were generated by chronic unpredictable mild stress

**FIGURE 1:**
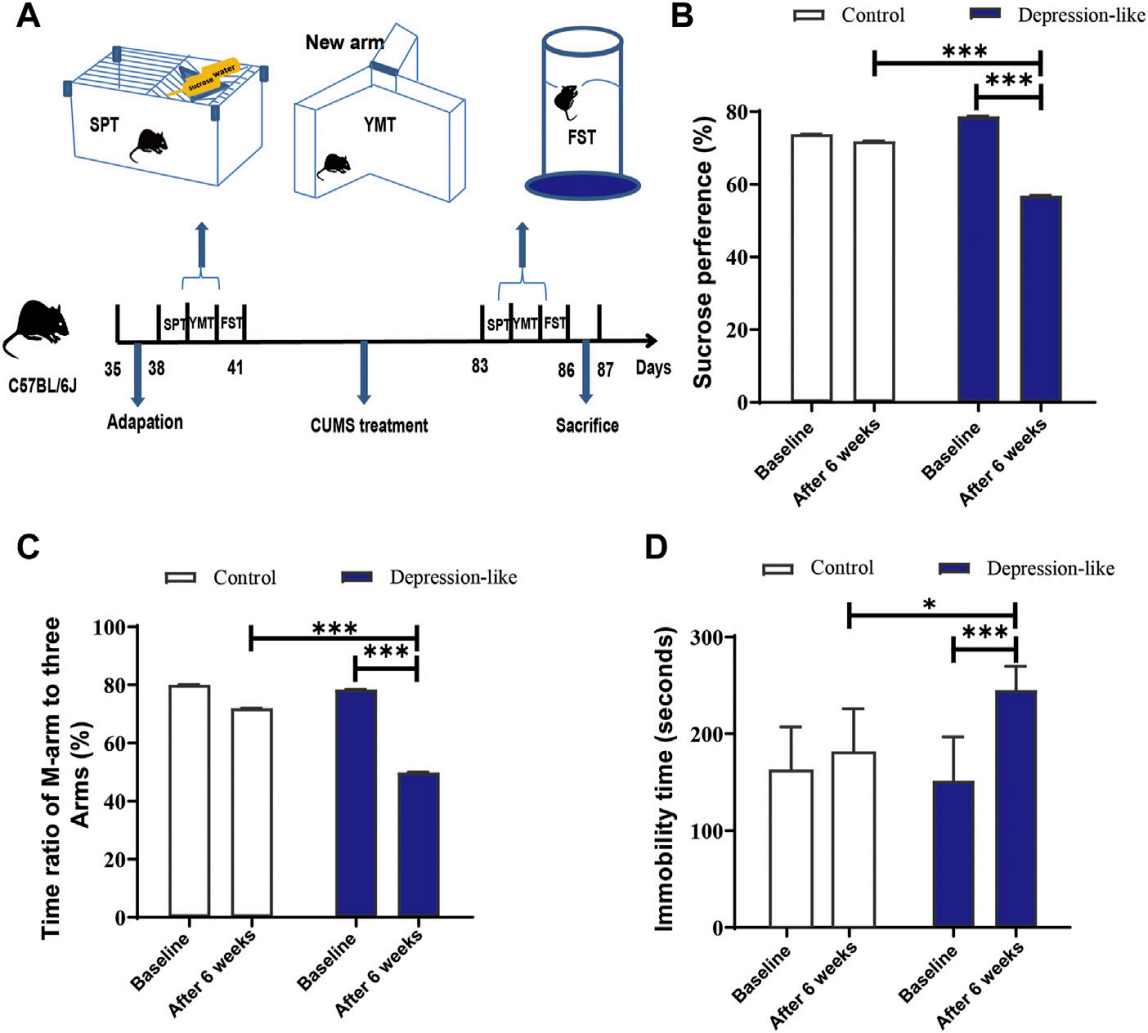
CUMS treatment leads mice to express depression-like behaviors. **(A)** Diagram of the production of depression-like mice. The procedure includes adaptation for a week, CUMS treatment for 6 weeks, behavioral tests, and definition. **(B)** SPT values (%) of the control and CUMS mice before and after treatment. **(C)** Time ratios of the M-arm to three arms before and after treatments in control and CUMS mice. **(D)** Immobile time of staying in the water cylinder before and after treatments in control and CUMS mice. The paired *t*-test was conducted for the comparisons before and after the CUMS treatment (***p < 0.001, **p < 0.01, and *p < 0.05). Control (*n* = 9) and depression-like (*n* = 14) mice were used to conduct this panel.

To produce mice with depression-like behaviors, female mice aged 35 days post-partum (dpp) were treated by CUMS or without stress for 6 weeks. After CUMS treatment, depression-like behaviors were assessed by behavioral tests (SPTs, YMTs, and FSTs). Since the three behavioral parameters of the treated mice varied so much, it was observed that only the mice that changed significantly in all three tests were classified as depression-like mice (Figure 1A). As shown in Figure 1B, after 6 weeks of treatment, the SPT values of the CUMS treatment mice were significantly lower than those mentioned earlier (56.88 ± 8.54% versus 78.79 ± 5.63%, *p* < 0.001, and *n* = 14). Also, the YMT values in the CUMS treatment group were also significantly lower than those mentioned earlier (49.91 ± 9.20% versus 78.43 ± 6.13%, *p* < 0.001, and *n* = 14) (Figure 1C). Significantly decreased FSTs values were also found, and the immobile time was 245.1 ± 24.86 s after CUMS treatment, while it was 151.6 ± 45.23 s before the CUMS treatments (*p* < 0.001, *n* = 14) (Figure 1D). A significant difference was also observed in such behavioral test values between the control and CUMS mice after 6 weeks (Figures 1B–D). According to the aforementioned behavioral tests, 14 mice out of a total of 70 met the criteria for depression. We considered these 14 mice as depression-like mice (20.00%, Table 1).

**TABLE 1:** CUMS-induced behavioral changes in the mice.

Test	Number	Percentage (%)
FST down more than 15%	38	54.29
YMT down more than 20%	36	51.43
Immobile time of FST in the last 5 min increased by more than 10%	60	85.71
Depression-like mice	14	20.00

### Clinical characteristics of ovarian insufficiency were observed in depression-like mice

**FIGURE 2:**
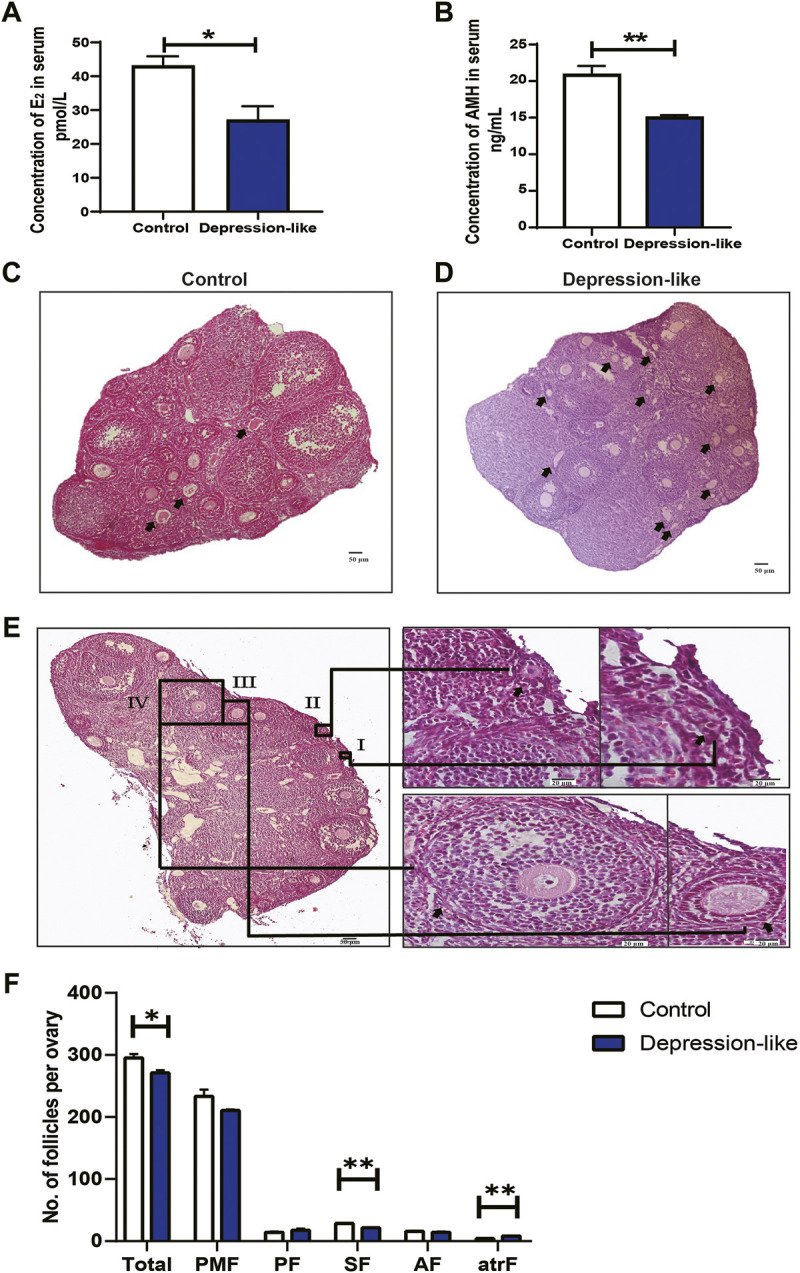
Quantification of ovarian follicles and measurement of serum hormone levels. **(A)** Sera collected from control (*n* = 4) and depression-like (*n* = 3) mice for hormone levels. Significantly reduced levels of E_2_ were observed in depression-like mice. **(B)** Significantly reduced levels of AMH were also observed in depression-like mice. **(C,D)** H&E staining of total ovarian sections of control (*n* = 3) and depression-like mice (*n* = 3). Prematurely active follicles that underwent atresia were noted (arrow). **(E)** H&E staining of typical morphologies of different-stage follicles. I, primordial follicle; II, primordial follicle, III, primary follicle; IV, secondary follicle; V, antral follicle. **(F)** Quantification of follicles in mouse ovaries of the control and depression-like groups. Abbreviations: total follicles (Total), primordial (PFM), primary (PF), secondary (SF), early antral (AF), and atretic follicles (atrF). Scale bars, 50 and 20 μm (enlargements) (**p < 0.01; *p < 0.05).

A growing body of research demonstrates that psychological stress in women is linked to ovarian developmental anomalies, including follicle and oocyte malformations (Zhai et al., 2020). But the mechanisms remain unclear. In order to know the effect of CUMS treatment on ovarian developments in female mice, serum hormone levels were measured in two groups of mice. The levels of E_2_ (*p* < 0.05, Figure 2A) and AMH (*p* < 0.01, Figure 2B) were significantly decreased in depression-like mice. Additionally, we found degraded oocytes were significantly piled up in depression-like mouse ovaries, suggesting more prematurely active follicles underwent atresia (Figures 2C,D, arrows). The typical morphologies of different-stage follicles including the primordial follicle, primary follicle, secondary follicle, and antral follicle were shown by H&E staining (Figure 2E). Then, total ovarian sections were conducted to compare the number of ovarian follicles, a remarkable decrease in total follicles was found (*p* < 0.05), and the number of second follicles in depression-like mouse ovaries was significantly lower (*p* < 0.01) (Figure 2F). These results suggest that mouse ovarian follicle development might be interrupted by CUMS treatment, and clinical characteristics of ovarian insufficiency in human patients were observed in depression-like mice.

### Increased granulosa cell apoptosis was observed in depression-like mouse ovaries

**FIGURE 3:**
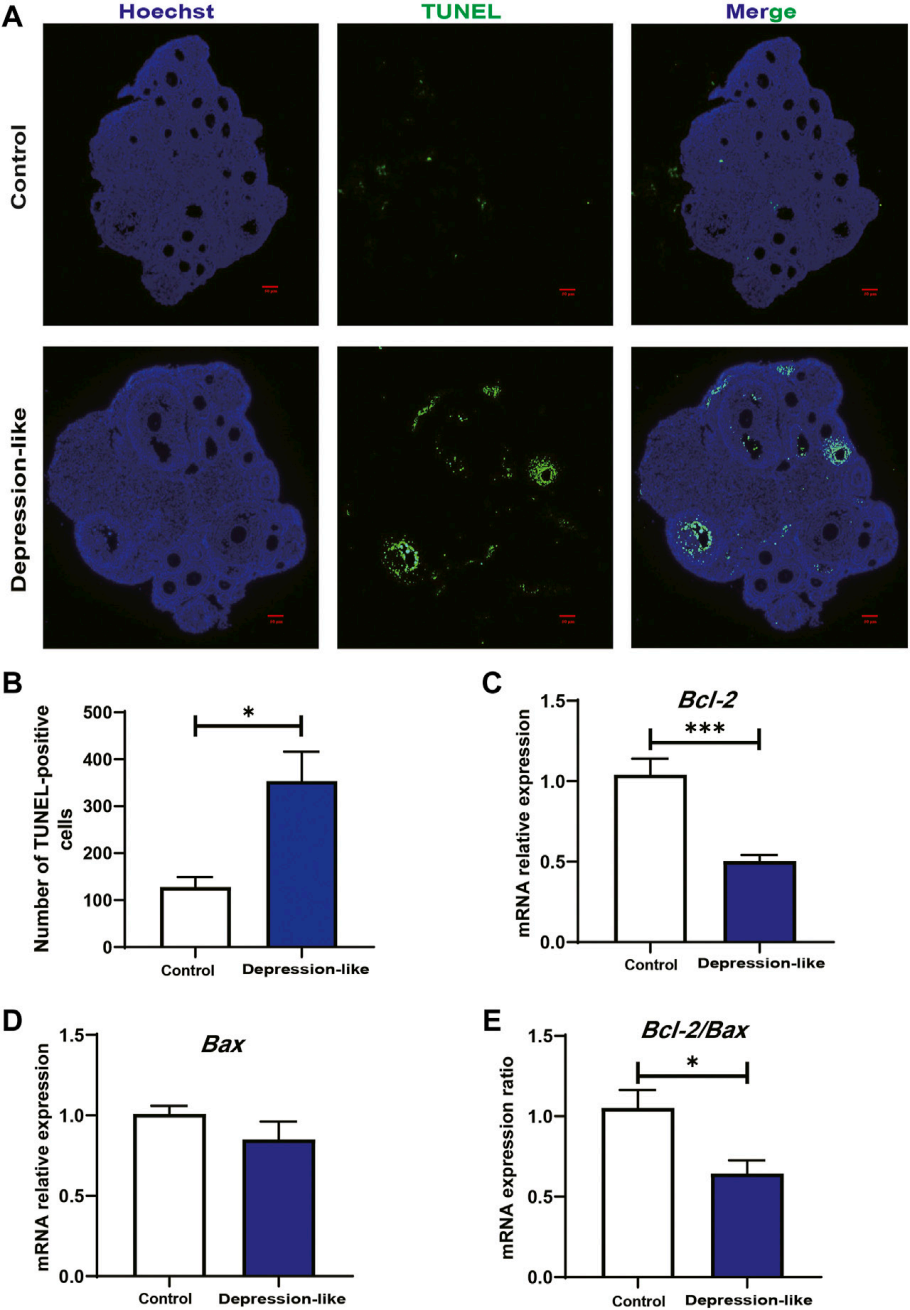
TUNEL staining of the ovarian section and relative mRNA levels of *Bcl2* and *Bax* in granulosa cells’ follicle development. **(A)** TUNEL assay showing the apoptosis of granulosa cells and atresia of follicles in control and depression-like ovarian tissues. Four mice in each group were detected. Green, TUNEL; blue, Hoechst. Scale bars, 50 μm. **(B)** Quantitative analysis of TUNEL-positive assays in the ovaries of the depression-like mice compared with control. **(C**,**D)** Relative mRNA levels of *Bcl-2* and *Bax* were performed by real-time PCR in GCs isolated from control and depression-like mice. **(E)** Quantification of mRNA levels of *Bcl-2/Bax* (**p < 0.01; *p < 0.05).

To test whether the reduction in serum E_2_ and ovary total follicles (especially the second follicles) in depression-like mice is caused by the apoptosis in granulosa cells, we first examined the ovary section by H&E staining. As shown in Figure 2C, degraded oocytes were significantly piled up in treated ovaries, suggesting more prematurely active follicles underwent atresia. TUNEL assays suggested that granulosa cells in the depression-like mouse ovaries did undergo more apoptosis (Figures 3A,B). For further confirmation, the expression of *Bcl2* and *Bax* in granulosa cells, which are markers of apoptosis, was performed by real-time PCR. As shown in Figures 3C–E, in comparison with the control mice, there was a significant difference in the expression of *Bcl2* and *Bcl2/Bax* in depression-like mice’s granulosa cells. These results indicate that the reduction in serum E_2_ and the defective follicle development in depression-like mice are associated with the apoptosis in granulosa cells.

### Overall composition of lipid classes altered in depression-like mouse ovaries

**FIGURE 4:**
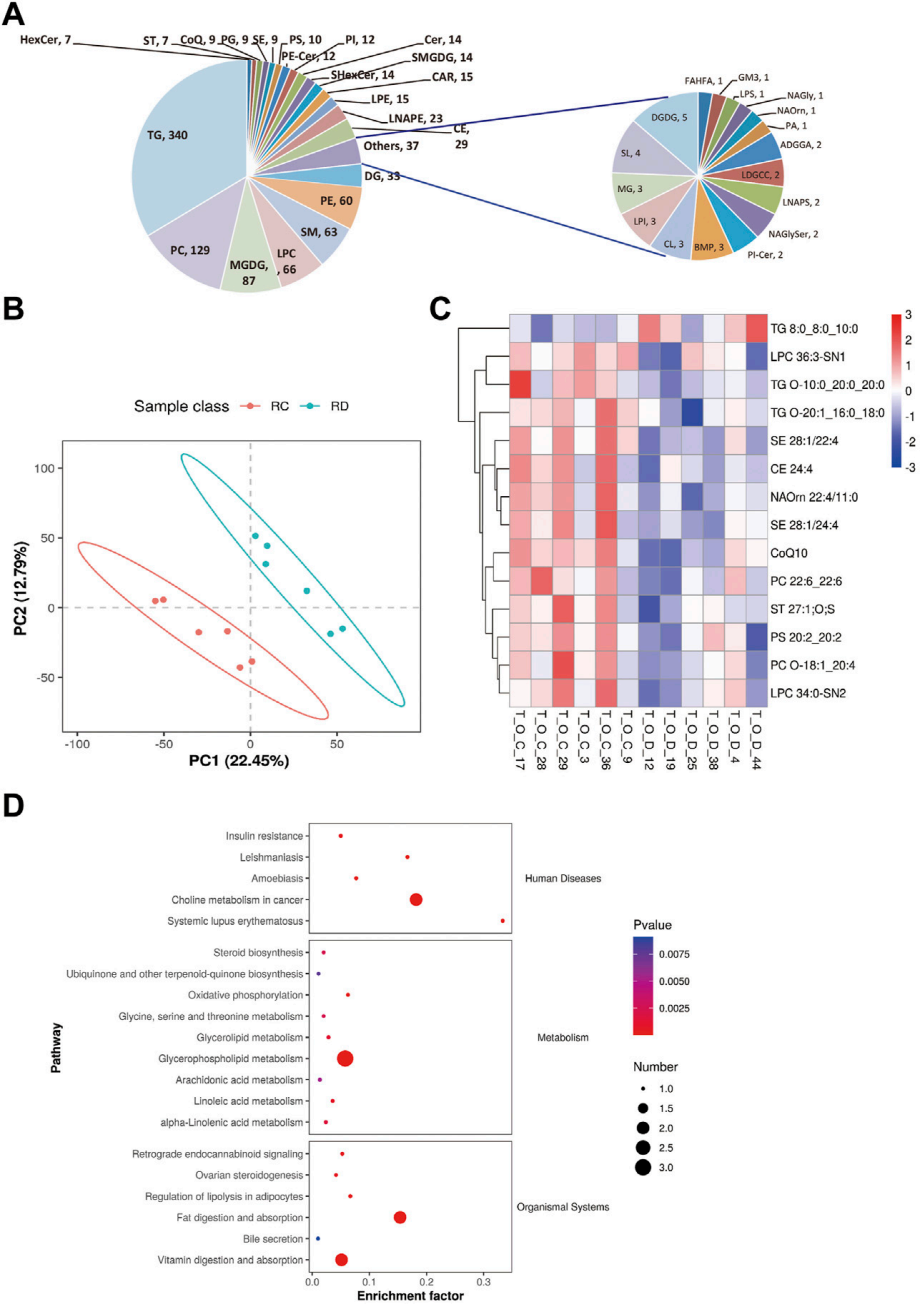
Changes in the overall lipid composition and distribution in the mouse ovary. **(A)** Lipids were extracted and analyzed, as described in the “Methods” section. Distribution of lipid classes that were considered for subsequent analysis in all of the samples detected by LC-MS/MS. **(B)** OPLS-DA plot of metabolomics separating depression-like and control ovarian samples. RC, control, and RD, depression-like. **(C)** Heat maps of differentially expressed metabolites in the ovaries of depression-like mice were significantly changed compared with control. T_O_C, control and T_O_D, depression-like. **(D)** KEGG enrichment analysis of differential metabolite changes in depression-like mice compared with control. Four ovaries in each group were detected.

To determine the changes in the overall lipid composition and distribution in ovaries in response to CUMS treatment, a non-targeted lipidomic strategy by liquid chromatography tandem mass spectrometry (LC-MS/MS) analysis was performed to reveal differential lipids between depression-like mice (*n* = 6) and controls (*n* = 6). We detected over 1,001 different lipid species in ovaries, consisting of 340 triacylglycerols (TGs), 129 phosphatidylcholines (PCs), 87 monogalactosyldiacylglycerols (MGDGs), 66 lysophophatidylcholines (LPCs), 63 sphingomyelins (SMs), 60 phosphatidylethanolamines (PEs), 33 diglycerides (DGs), and 223 other lipid classes (Figure 4A). The abbreviations of these quantified lipid classes are shown in Supplementary Table S2. The orthogonal projections to latent structure discriminant analysis (OPLS-DA) plot shows a clear separation of two classes (depression-like vs. control, Figure 4B), in details R2 = 0.97 and Q2 = −0.51 (Supplementary Figure S1). From the OPLS-DA model, 14 features were selected that differed between the two groups with variables of importance for the projection of VIP (variable importance in projection) > 1.0, *p* < 0.05, and FC (fold change) ≥ 1.5 (or FC ≤ 0.67) (Figure 4C; Supplementary Table S3). We found that CUMS treatment significantly decreased the contents of the following lipid metabolisms in the ovary, including LPC (36:3-SN1 and 34:0-SN2), TG (O-10:0_20:0_20:0 and O-20:1_16:0_18:0), campesterol ester (SE 28:1/22:4 and SE 28:1/24:4), cholesteryl ester (CE 24:4), N-acyl ornithine (NAOrn), coenzyme Q 10 (CoQ10), PC (22:6_22:6 and O-18:1_20:4), sterol sulfate (ST 27:1; O; S), and phosphatidylserine (PS 20:2_20:2) (Figure 4C). However, the increased level of TAG (8:0_8:0_10:0) in the ovaries of depression-like mice was related to fat digestion and absorption, vitamin digestion and absorption, and insulin resistance pathways (Supplementary Figure S2–4). Additionally, CE is supported by the ovarian steroidogenesis pathway (Supplementary Figure S5), and the decreased level of CE might be associated with the decreased level of E_2_ in the serum (Figure 4D). These results suggest that the overall composition of lipid classes was altered in the depression-like mouse ovaries.

### Cell death and growth-related pathways were impaired in depression-like mouse ovaries

**FIGURE 5:**
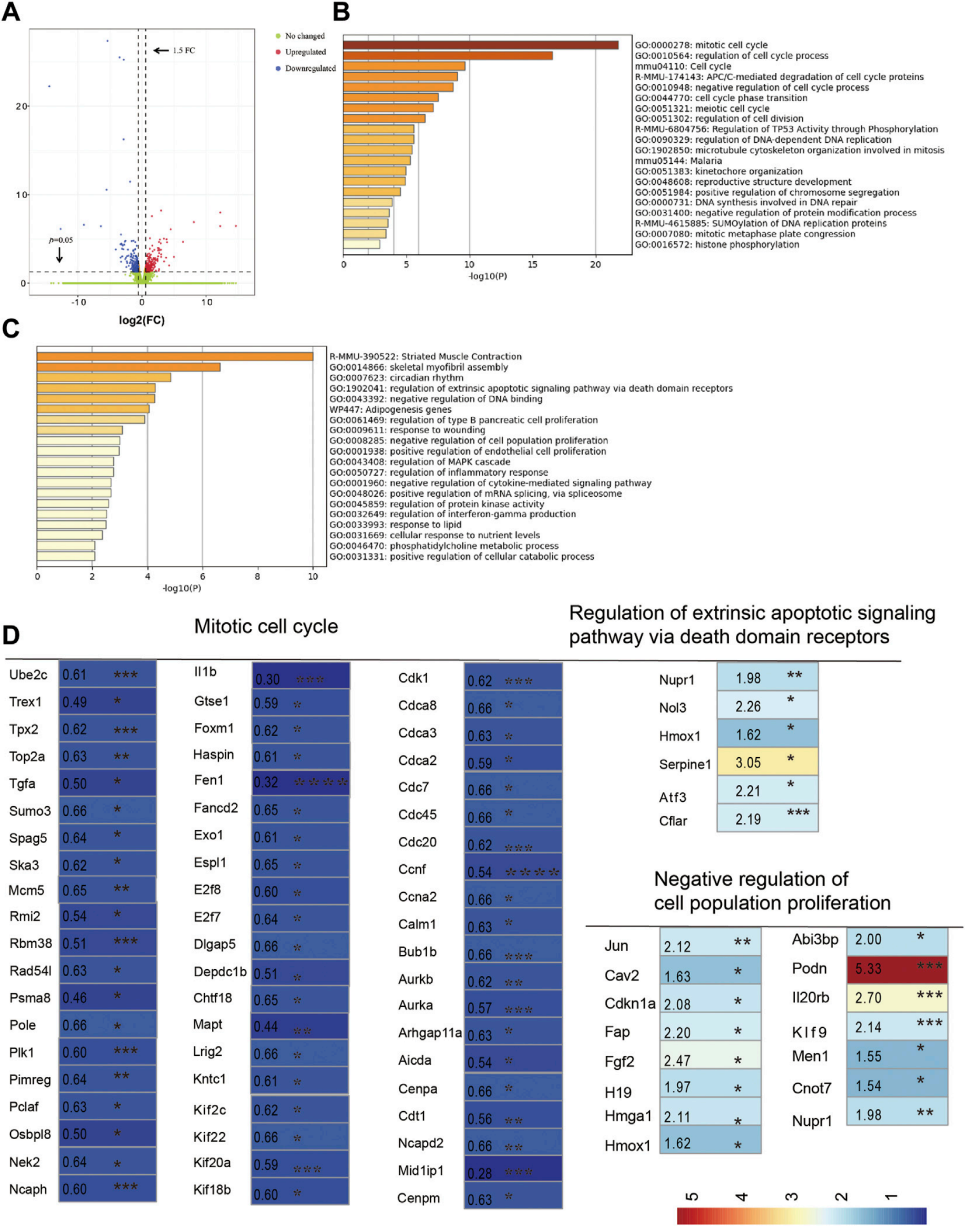
RNA sequencing analysis of ovaries. **(A)** Volcano plot showed variance in gene expression with regard to fold change and *p*-value. **(B)** Pathway and process enrichment analysis of downregulated gene lists (211) in depression-like mouse ovaries, colored by *p*-values. **(C)** Pathway and process enrichment analysis of upregulated gene lists (212) in depression-like mouse ovaries, colored by *p*-values. **(D)** Heat map was built by a panel of 80 differentially expressed genes, which were annotated in GO terms of the mitotic cell cycle, regulation of the extrinsic apoptotic signaling pathway *via* death domain receptors, and negative regulation of cell population proliferation (***p < 0.001, **p < 0.01, and *p < 0.05). Student’s *t*-test was used for the comparisons between depression-like (*n* = 3) and control mice (*n* = 3).

To further understand how CUMS treatment affects follicle development, mouse ovaries from the control and depression-like groups were collected for RNA-seq. The total number of reads generated from each sample varied from 40218254 to 52603858, which were in a unique manner, with mapping rates between 97.81 and 97.89 (Supplementary Table S4). In total, at least 24,838 genes were expressed in the control and CUMS-treated samples. Among them, 423 significantly differentially expressed genes (DEGs) and 212 upregulated and 211 downregulated DEGs (fold change, FC ≥ 1.5 or FC ≤ 0.67, *p* < 0.05, depression-like/control) were identified (Figure 5A; Supplementary Table S1). Pathway and process enrichment analysis was performed with these DEGs, and the results showed that the downregulated genes were enriched in gene ontology annotations of the mitotic cell cycle, regulation of cell cycle processes, and pathway annotations of the cell cycle (Figure 5B). However, the upregulated genes were annotated as being involved in striated muscle contraction, skeletal myofibril assembly, circadian rhythm, and regulation of the extrinsic apoptotic signaling pathway (Figure 5C). Notably, our results showed that 60 genes associated with the GO:0000278 mitotic cell cycle were found to be significantly decreased in the CUMS-treated ovaries (Figure 5D; Supplementary Table S5). Five genes associated with the GO:1902041 regulation of the extrinsic apoptotic signaling pathway *via* death domain receptors and 15 genes associated with GO:0008285 negative regulation of cell population proliferation were found to be significantly increased in the depression-like mouse ovaries (Figure 5D; Supplementary Table S6). Then, real-time PCR was employed to confirm the validity of RNA-seq analysis. Two downregulated cell cycle-associated genes (*Il1b* and *Tgfa*) and three upregulated cell population proliferation-associated genes (*Fgf2*, *Cdkn1a*, and *Serpine 1*) were selected to amplify. These results indicated a good agreement between RNA-seq data and real-time PCR (Supplementary Figure S6). Taken together, cell death and growth-related pathways (including the mitotic cell cycle, regulation of the extrinsic apoptotic signaling pathway *via* death domain receptors, and negative regulation of cell population proliferation) were likely implicated in the defective follicle development of depression-like mice.

## Discussion

Stress is an unavoidable part of our lives. It is known to have a negative impact on a person’s physical and mental health by interfering with the proper functioning of numerous systems, including the reproductive system. Several research studies have been conducted with the goal of developing an animal model for evaluating the relationship between chronic stress and early ovarian insufficiency (ovarian insufficiency) (Fu et al., 2018; Fu et al., 2020; Xu et al., 2020). Although similar to the aforementioned studies, the CUMS method was developed in a mouse model to evaluate the impact of psychological stress on ovary follicle development in this study. After CUMS treatment for 6 weeks, degraded oocytes were significantly piled up in the treated ovaries, suggesting more prematurely active follicles underwent atresia (Figure 2C, arrows). Additionally, a remarkable decrease in total ovarian follicles was found, especially the second follicles, which suggested that mouse ovarian follicle development might be interrupted by CUMS treatment (Figures 2D,F). Moreover, the decreased levels of E_2_ and AMH in depression-like mice (Figures 2A,B) were in accordance with those reported by Fu et al. (2018) and Li et al. (2021). Taken together, the alterations in hormonal levels and defective follicle development demonstrated that CUMS-treated mice exhibit ovarian dysfunction similar to ovarian insufficiency patients. Also, a mouse model to evaluate the impact of psychological stress on ovary follicle development was successfully built.

Cholesterol has an important influence on mammalian follicular development, through regulating ovarian sex hormone synthesis as a key substrate (Huang et al., 2019). LC-MS/MS analysis was conducted to know the changes in lipid composition and distribution in ovaries in response to CUMS treatment, and the abundance of cholesteryl ester was significantly decreased (Figure 4C), which might be associated with the decreased level of E_2_ in the serum (Figure 2A). Additionally, the destruction of lipid homeostasis in the mouse ovary might be caused by high amounts of glucocorticoids released, which have been reported to be involved in stimulating the production of progestins in a manner suggestive of the accumulation of lipids, especially cholesterol esters (Towns et al., 1999). Furthermore, the increased level of TAG (8:0_8:0_10:0) in the ovaries of depression-like mice was related to fat digestion and absorption, vitamin digestion and absorption, and insulin resistance pathways (Supplementary Figure S2, S3, S5). These aforementioned results suggest that psychological stress is harmful to female ovary function by interrupting lipid homeostasis. Also, there might be a certain causal relationship between the lipid homeostasis disruption and ovarian insufficiency.

Glucocorticoids reduce oocyte developmental potential by inducing ovarian cell death (Yuan et al., 2016). In our study, granulosa cells did undergo more apoptosis (Figures 3A,B), and the expression of Bcl2 and Bcl2/Bax in granulosa cells was significantly decreased in depression-like mouse ovaries (Figures 3C,D). Then, total transcriptome analysis in the mouse ovary was conducted to understand how CUMS treatment affects follicle development. Notably s, our result showed that 60 genes associated with the mitotic cell cycle were found to be significantly decreased in depression-like mouse ovaries (Figure 5D; Supplementary Table S5). A total of five genes associated with the regulation of extrinsic apoptotic signaling pathways *via* death domain receptors and 15 genes associated with negative regulation of cell population proliferation were found to be significantly increased in depression-like mouse ovaries (Figure 5D; Supplementary Table S6). These aforementioned data indicated that cell death and growth are likely implicated in the defective follicle development of CUMS-treated mice.

In summary, in order to mimic the ovary environment under psychological stress, C57BL/6J female mice were subjected to CUMS, and then, depression-like mice were selected and identified according to the behavioral tests. Lipid homeostasis of the ovary was detected in our study to explain the underline mechanism, and the decreased abundance of CE was supported to be associated with the downregulated E_2_. Moreover, granulosa cells did undergo more apoptosis in response to psychological stress, which was caused by Bcl2 and Bcl2/Bax downregulated in granulosa cells and related to the disorder of cell death and growth in the ovary. This study will provide a better understanding of the female reproductive problem under psychological stress.

## Data Availability

The raw data supporting the conclusion of this article will be made available by the authors, without undue reservation.
