# Supplementary material for: Chronic unpredictable mild stress-induced mouse ovarian insufficiency by interrupting lipid homeostasis in the ovary
Source: Front Cell Dev Biol. 2022 Sep 8;10:933674. doi: 10.3389/fcell.2022.933674 (PMC9493201; doi:10.3389/fcell.2022.933674)
Supplement: Supplementary file 2 [file DataSheet2.docx]

| **Gene** | **Forward primer (5’-3’)** | **Reverse primer (3’-5’)** | **Annealing temperature (℃)** | **Fragment size(bp)** |
| --- | --- | --- | --- | --- |
| Gapdh | GGCAAATTCAACGGCACAGT | GGCCTCACCCCATTTGATGT | 60 | 111 |
| Bax | GAGACACCTGAGCTGACCTT | GCTCCATATTGCTGTCCAGT | 60 | 106 |
| Bcl-2 | CCGGGAGAACAGGGTATGAT | AGGCTGGAAGGAGAAGATGC | 60 | 149 |
| Mapt | GTCCTCGCCTTCTGTCGATT | GCTGTGGGGGAGACTCTTTT | 60 | 153 |
| Tgfa | ATCCTGTTAGCTGTGTGCCA | GGAATCTGGGCACTTGTTGA | 60 | 105 |
| Fgf2 | GGCTGCTGGCTTCTAAGTGT | TCTGTCCAGGTCCCGTTTTG | 60 | 162 |
| Cdkn1a | CCGTTGTCTCTTCGGTCCC | CATGAGCGCATCGCAATC | 60 | 61 |
| Serpine1 | AGGCACTGCAAAAGGTCAGG | AGAGGATTGTCTCTGTCGGG | 60 | 159 |
| Il1b | TGCCACCTTTTGACAGTGATG | AAGGTCCACGGGAAAGACAC | 60 | 220 |

**Table S1. Primers used for Real-time PCR analysis.**

**Table S2. Abbreviation of the quantified lipid classes.**

| **Abbrev.** | **Lipid class** | **Abbrev.** | **Lipid class** |
| --- | --- | --- | --- |
| TG | Triacylglycerol | LPE | Lysophosphatidylethanolamine |
| STSE | Stigmasterol ester | PI_Cer | Ceramide phosphoinositol |
| SSulfate | Sterol sulfate | PI | Phosphatidylinositol |
| SM | Sphingomyelin | LDGCC | Lysodiacylglyceryl-3-O-carboxyhydroxymethylcholine |
| SL | Sulfonolipid | PE_Cer | Ceramide phosphoethanolamine |
| SHexCer | Sulfatide | PE | Phosphatidylethanolamine |
| PC | Phosphatidylcholine | LNAPS | N-acyl-lysophosphatidylserine |
| NAGlySer | N-acyl glycyl serine | EtherPE | Ether-linked phosphatidylethanolamine |
| PG | Phosphatidylinositol | MGDG | Monogalactosyldiacylglycerol |
| LPS | Lysophosphatidylserine | LPI | Lysophosphatidylinositol |
| NAOrn | N-acyl ornithine | FAHFA | Fatty acid ester of hydroxyl fatty acid |
| MG | Monoacylglycerol | LNAPE | N-acyl-lysophosphatidylethanolamine |
| CoQ | Coenzyme Q | HexCer_NS | Hexosylceramide non-hydroxyfatty acid-sphingosine |
| GM3 | Ganglioside GM3 | EtherMGDG | Ether-linked monogalactosyldiacylglycerol |
| PS | Phosphatidylserine | EtherTG | Ether-linked triacylglycerol |
| EtherSMGDG | Semino lipid | EtherPG | Ether-linked phosphatidylglycerol |
| CAR | Acylcarnitine | EtherPC | Ether-linked phosphatidylcholine |
| NAGly | N-acyl glycine | EtherLPE | Ether-linked lysophosphatidylethanolamine |
| LPC | Lysophophatidylcholine | EtherDGDG | Ether-linked digalactosyldiacylglycerol |
| DG | Diacylglycerol | Cer_NDS | Ceramide non-hydroxyfatty acid-dihydrosphingosine |
| CL | Cardiolipin | Cer_NS | Ceramide non-hydroxyfatty acid-sphingosine |
| CE | Cholesteryl ester | HexCer_HS | Hexosylceramide hydroxyfatty acid-sphingosine |
| CASE | Campesterol ester | EtherLPC | Ether-linked lysophosphatidylcholine |
| BRSE | Brassicasterol ester | BMP | Bismonoacylglycerophosphate |
| AHexSTS | Acylhexosyl stigmasterol | AHexCAS | Acylhexosyl campesterol |
| ADGGA | Acyl diacylglyceryl glucuronide |  |  |

**Table S3. Details of 14 differential features between the two groups.**

| **t_R_(min)** | **Metabolite** | **Formula** | **Measured m/z** | **ratio** | **regulation** |
| --- | --- | --- | --- | --- | --- |
| 3.49 | ST 27:1;O;S | C_27_H_46_O_4_S | 465.3032[M-H]- | 0.64 | down |
| 5.82 | TG 8:0/10:0 | C_29_H_54_O_6_ | 521.3758[M+Na]+ | 1.61 | up |
| 6.50 | PC 22:6 | C_52_H_80_NO_8_P | 878.5797[M+H]+ | 0.63 | down |
| 7.00 | LPC 36:3-SN1 | C_44_H_84_NO_7_P | 770.5925[M+H]+ | 0.64 | down |
| 7.03 | PC O-18:1_20:4 | C_46_H_84_NO_7_P | 838.5926[M+HCOO]- | 0.62 | down |
| 7.06 | PS 20:2 | C_46_H_82_NO_10_P | 840.5689[M+H]+ | 0.65 | down |
| 7.08 | NAOrn 22:4/11:0 | C_38_H_66_N_2_O_5_ | 631.5019 [M+H]+ | 0.57 | down |
| 7.83 | LPC 34:0-SN2 | C_42_H_86_NO_7_P | 748.6181[M+H]+ | 0.60 | down |
| 9.25 | CoQ10 | C_59_H_90_O_4_ | 863.6840[M+H]+ | 0.66 | down |
| 10.12 | SE 28:1/22:4 | C_50_H_82_O_2_ | 732.6616 [M+NH4]+ | 0.56 | down |
| 10.26 | CE 24:4 | C_51_H_84_O_2_ | 746.6786[M+NH4]+ | 0.67 | down |
| 10.34 | SE 28:1/24:4 | C_52_H_86_O_2_ | 760.6856 [M+NH4]+ | 0.59 | down |
| 10.38 | TG O-20:1_16:0_18:0 | C_57_H_110_O_5_ | 897.8200[M+Na]+ | 0.62 | down |
| 10.49 | TG O-10:0_20:0_20:0 | C_53_H_104_O_5_ | 843.7656[M+Na]+ | 0.63 | down |

**Table S4. Sample information for RNA-seq.**

| **Sample** | **Raw Data** | | **Valid Data** | | **Valid Ratio(reads)** | **Q30%** | **GC content%** |
| --- | --- | --- | --- | --- | --- | --- | --- |
|  | **Reads** | **Base** | **Reads** | **Base** |  |  |  |
| T_O_C_17 | 52603858 | 7.89G | 51493816 | 7.72G | 97.89 | 98.37 | 50.00 |
| T_O_C_3 | 44576422 | 6.69G | 43633026 | 6.54G | 97.88 | 97.94 | 50.50 |
| T_O_C_9 | 48012968 | 7.20G | 46995788 | 7.05G | 97.88 | 98.18 | 49.50 |
| T_O_D_12 | 48423780 | 7.26G | 47375436 | 7.11G | 97.84 | 97.91 | 49.00 |
| T_O_D_38 | 40218254 | 6.03G | 39337276 | 5.90G | 97.81 | 98.18 | 49.50 |
| T_O_D_44 | 50163886 | 7.52G | 49095514 | 7.36G | 97.87 | 97.96 | 49.00 |

Note: Control group include T_O_C_17, T_O_C_3 and T_O_C_9. Depression-like group include T_O_D_12, T_O_D_38 and T_O_D_44.

**Table S5. Top 10 clusters with their representative enriched terms (one per cluster) of the down-regulated genes in depression-like mice ovaries.**

| **GO** | **Category** | **Description** | **Count (%)** | **Log10 (P)** | **Log10 (q)** | **Genes** |
| --- | --- | --- | --- | --- | --- | --- |
| GO:0000278 | GO Biological Processes | mitotic cell cycle | 35(22.12) | -21.78 | -17.57 | Bub1b,Calm1,Ccna2,Ccnf,Cdk1,Cdc45,Cenpa,Foxm1,Haspin,Nek2,Plk1,Pole,Kif20a,Aurkb,Aurka,Trex1,Cdca8,E2f7,Spag5,Rbm38,Cdt1,Ncapd2,Ube2c,Kif18b,Tpx2,Kif2c,Espl1,Cdc20,E2f8,Kif22,Kntc1,Fancd2,Ncaph,Dlgap5,Ska3,Cdc7,Rad54l,Top2a,Pclaf,Chtf18,Mcm5,Exo1,Gtse1,Cenpm,Psma8,Rmi2,Cdca3,Cdca2,Pimreg,Fen1,Il1b,Tgfa,Mapt,Mid1ip1,Depdc1b,Arhgap11a,Sumo3,Osbpl8,Aicda,Lrig2 |
| GO:0010564 | GO Biological Processes | regulation of cell cycle process | 33(20.89) | -16.55 | -12.89 | Aicda,Bub1b,Calm1,Ccnf,Cdk1,Cdc7,Cdkn2d,Fen1,Haspin,Il1b,Nek2,Plk1,Kif20a,Rrm2,Aurkb,Aurka,Tgfa,Trex1,E2f7,Spag5,Rbm38,Cdt1,Ncapd2,Ube2c,Tpx2,Psma8,Cdc20,Cdca2,E2f8,Kntc1,Fancd2,Ncaph,Rmi2,Mapt |
| mmu04110 | KEGG Pathway | Cell cycle | 12(7.59) | -9.64 | -6.60 | Bub1b,Ccna2,Cdk1,Cdc45,Cdc7,Cdkn2d,Mcm5,Plk1,Gadd45g,Espl1,Cdc20,Tfdp2,Fen1,Foxm1,Pole,Rad54l,Top2a,Trex1,Exo1,E2f7,Rbm38,Pclaf,Kif22,Fancd2,Rmi2,Ankle1,Rrm2,Cdt1,Chtf18,Aicda,Gtse1,Psma8,Ube2c |
| R-MMU-174143 | Reactome Gene Sets | APC/C-mediated degradation of cell cycle proteins | 10(6.33) | -9.04 | -6.06 | Bub1b,Ccna2,Cdk1,Nek2,Plk1,Aurkb,Aurka,Ube2c,Psma8,Cdc20,Haspin,Kif22,Calm1,Espl1,Sumo3,Pbk |
| GO:0010948 | GO Biological Processes | negative regulation of cell cycle process | 15(9.49) | -8.69 | -5.73 | Bub1b,Ccnf,Cdk1,Cdkn2d,Haspin,Nek2,Plk1,Aurkb,Trex1,E2f7,Rbm38,Cdt1,E2f8,Kntc1,Fancd2,Cdc7,Il1b,Rrm2,Tgfa,Ube2c,Cdca2,Dlgap5,Aurka,Foxm1 |
| GO:0044770 | GO Biological Processes | cell cycle phase transition | 11(6.96) | -7.52 | -4.77 | Bub1b,Calm1,Ccna2,Ccnf,Cdk1,Cdc7,Foxm1,Plk1,Pole,E2f7,Ube2c,Nek2,Aurka,Gtse1,Tpx2,Psma8,Amh,Haspin,Melk,Snrk,Aurkb,Map4k1,Pbk,Taf4,Kif22,Mgat1,Nfya,Padi2,Sumo3,Zfp451,Pclaf |
| GO:0051321 | GO Biological Processes | meiotic cell cycle | 14(8.86) | -7.13 | -4.40 | Bub1b,Nek2,Plk1,Rad54l,Aurka,Top2a,Exo1,Ncapd2,Mapk1ip1,Psma8,Espl1,Fancd2,Chtf18,Ncaph,Amh,Tnfaip6,Dhh,Arrb1,Dnd1 |
| GO:0051302 | GO Biological Processes | regulation of cell division | 11(696) | -6.45 | -3.87 | Calm1,Il1b,Plk1,Kif20a,Aurkb,Aurka,Tgfa,Thbs4,E2f7,Kif18b,E2f8,Ccna2,Cdk1,Tmed2,Socs3,Foxm1 |
| R-MMU-6804756 | Reactome Gene Sets | Regulation of TP53 Activity through Phosphorylation | 7(4.43) | -5.60 | -3.10 | Ccna2,Aurkb,Aurka,Exo1,Tpx2,Rmi2,Taf4,Cdk1,E2f7,E2f8,Pole,Sumo3,Pclaf,Fancd2,Trim25,Ppp4r2 |
| GO:0090329 | GO Biological Processes | regulation of DNA-dependent DNA replication | 6(3.8) | -5.60 | -3.10 | Aicda,Cdc7,E2f7,Cdt1,E2f8,Chtf18,Ccna2,Cdk1,Rrm2,Aurka,Ube2c |

"Count" is the number of genes in the user-provided lists with membership in the given ontology term. "%" is the percentage of all of the user-provided genes that are found in the given ontology term (only input genes with at least one ontology term annotation are included in the calculation). "Log10(P)" is the p-value in log base 10. "Log10(q)" is the multi-test adjusted p-value in log base 10.

**Table S7. Top 10 clusters with their representative enriched terms (one per cluster) of the up-regulated genes in depression-like mice ovaries.**

| **GO** | **Category** | **Description** | **Count (%)** | **Log10**  **(P)** | **Log10**  **(q)** | **Genes** |
| --- | --- | --- | --- | --- | --- | --- |
| R-MMU-390522 | Reactome Gene Sets | Striated Muscle Contraction | 8(5) | -10.00 | -5.73 | Acta1,Actc1,Actn2,Myh3,Myh8,Tnnc2,Tnni2,Tnnt3,Mylpf,Ttn,Map2k3,Kcnd3,Slmap,Mybpc1,Cflar,Shank3,Stc1,Tead1,Nol3 |
| GO:0014866 | GO Biological Processes | skeletal myofibril assembly | 4(2.5) | -6.62 | -3.05 | Acta1,Actc1,Cflar,Ttn,Actn2,Tnnt3,Trpm7,Cfd,Atf3,Cav2,Mylpf,Wnt7b,Nupr1,Nol3,Zbtb42,Cirbp,Cnot7,Haus2,Ythdf3,Fgf2,Ccr7,Myh8,Kcnd3,Coro6,Ppp1r9a,Septin2,Pla2g5,Shank3,Camsap2 |
| GO:0007623 | GO Biological Processes | circadian rhythm | 8(5) | -4.85 | -1.63 | Dbp,Id4,Ncoa2,Per1,Per3,Nr1d1,Kdm2a,Nr1d2,Klf9,Tef,Nr1h4 |
| GO:1902041 | GO Biological Processes | regulation of extrinsic apoptotic signaling pathway via death domain receptors | 5(3.12) | -4.29 | -1.25 | Atf3,Cflar,Hmox1,Serpine1,Nol3,Nupr1 |
| GO:0043392 | GO Biological Processes | negative regulation of DNA binding | 5(3.12) | -4.25 | -1.24 | Ddit3,Hmox1,Jun,Mdfi,Cpne1,Atf3,Cdkn1a,Fabp4,Nek7 |
| WP447 | WikiPathways | Adipogenesis genes | 7(4.38) | -4.06 | -1.10 | Cfd,Fabp4,Ddit3,Hmga1,Ncoa2,Serpine1,Scd1,Lamb3 |
| GO:0061469 | GO Biological Processes | regulation of type B pancreatic cell proliferation | 3(1.88) | -3.90 | -0.98 | Men1,Nupr1,Nr1d1 |
| GO:0009611 | GO Biological Processes | response to wounding | 10(6.25) | -3.08 | -0.36 | Cdkn1a,Cflar,Ddit3,Fgf2,Hmox1,Ccn1,Jun,Timp1,Nol3,Mir22hg,Nupr1 |
| GO:0008285 | GO Biological Processes | negative regulation of cell population proliferation | 15(9.38) | -2.98 | -0.30 | Cav2,Cdkn1a,Fap,Fgf2,H19,Hmga1,Hmox1,Jun,Klf9,Men1,Cnot7,Nupr1,Il20rb,Podn,Abi3bp |
| GO:0001938 | GO Biological Processes | positive regulation of endothelial cell proliferation | 5(3.12) | -2.97 | -0.30 | Cav2,Fgf2,Hmox1,Jun,Nras,Cflar,Klf9,Men1,Nupr1,Nr1d1,Map2k3,Cdkn1a,Serpine1,Cbl,Wnt7b,Ddit3,Pla2g5,Hspa1a,Dusp8 |

"Count" is the number of genes in the user-provided lists with membership in the given ontology term. "%" is the percentage of all of the user-provided genes that are found in the given ontology term (only input genes with at least one ontology term annotation are included in the calculation). "Log10(P)" is the p-value in log base 10. "Log10(q)" is the multi-test adjusted p-value in log base 10.
