# Supplementary material for: Chronic unpredictable mild stress-induced mouse ovarian insufficiency by interrupting lipid homeostasis in the ovary
Source: Front Cell Dev Biol. 2022 Sep 8;10:933674. doi: 10.3389/fcell.2022.933674 (PMC9493201; doi:10.3389/fcell.2022.933674)
Supplement: Supplementary file 3 [file DataSheet1.docx]

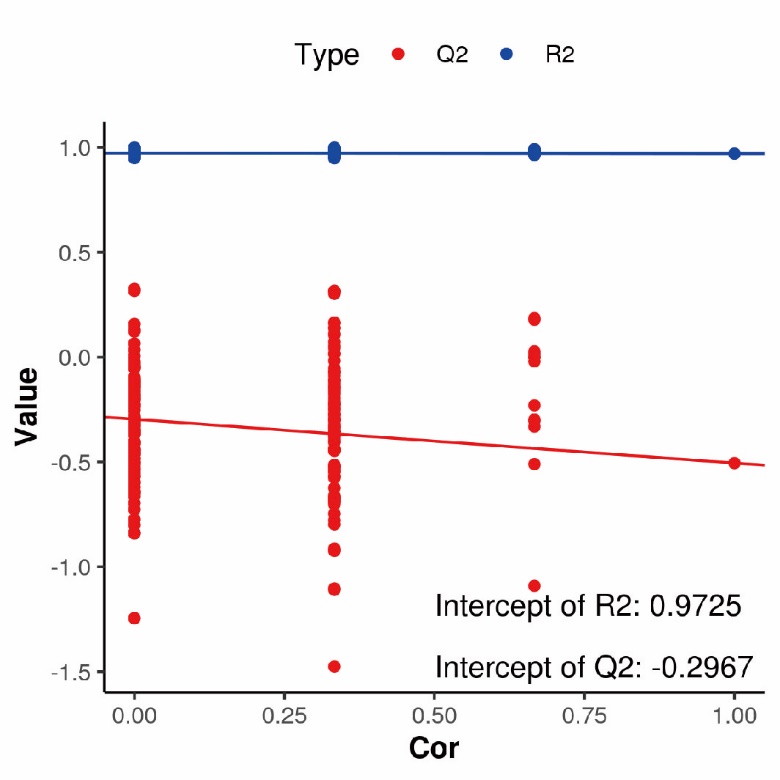


**Figure S1. Validation of PLSDA between the control and depression-like group.**


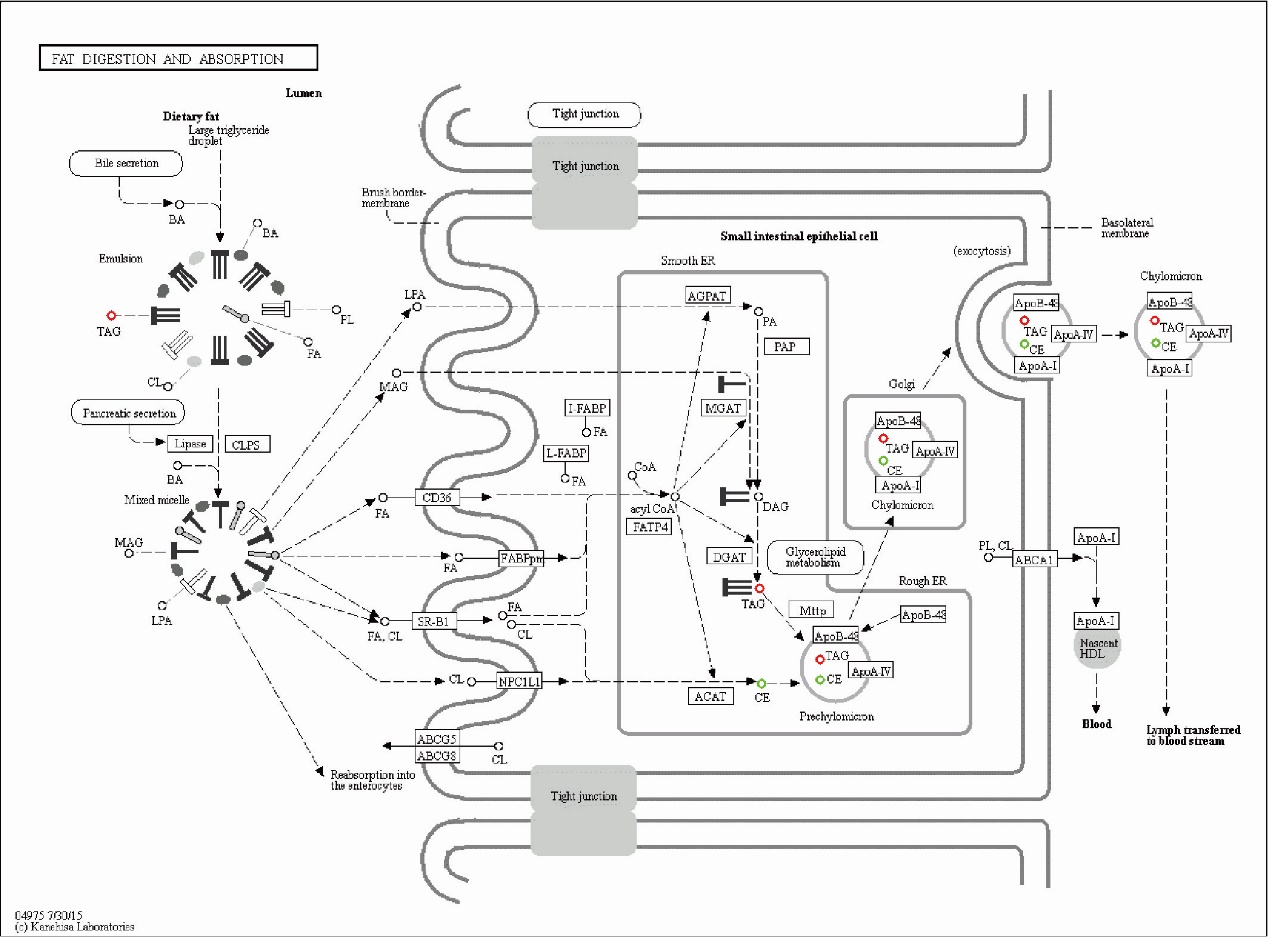


**Figure S2. Details of digestion and absorption pathway. C.** Note of vitamin digestion and absorption pathway. **D.** Note of insulin resistance pathway. **E.** Note of ovarian steroidogenesis pathway.


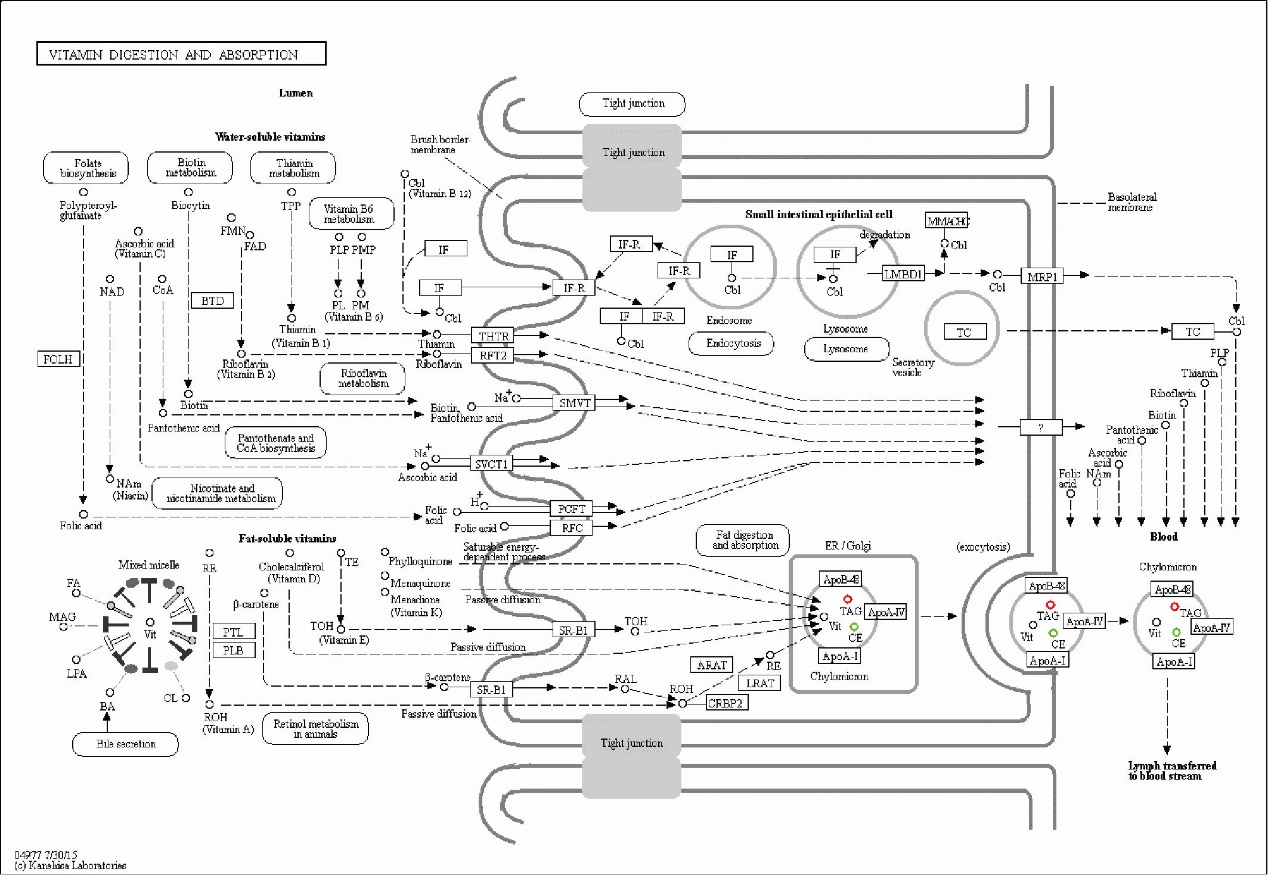


**Figure S3. Details of vitamin digestion and absorption pathway.**


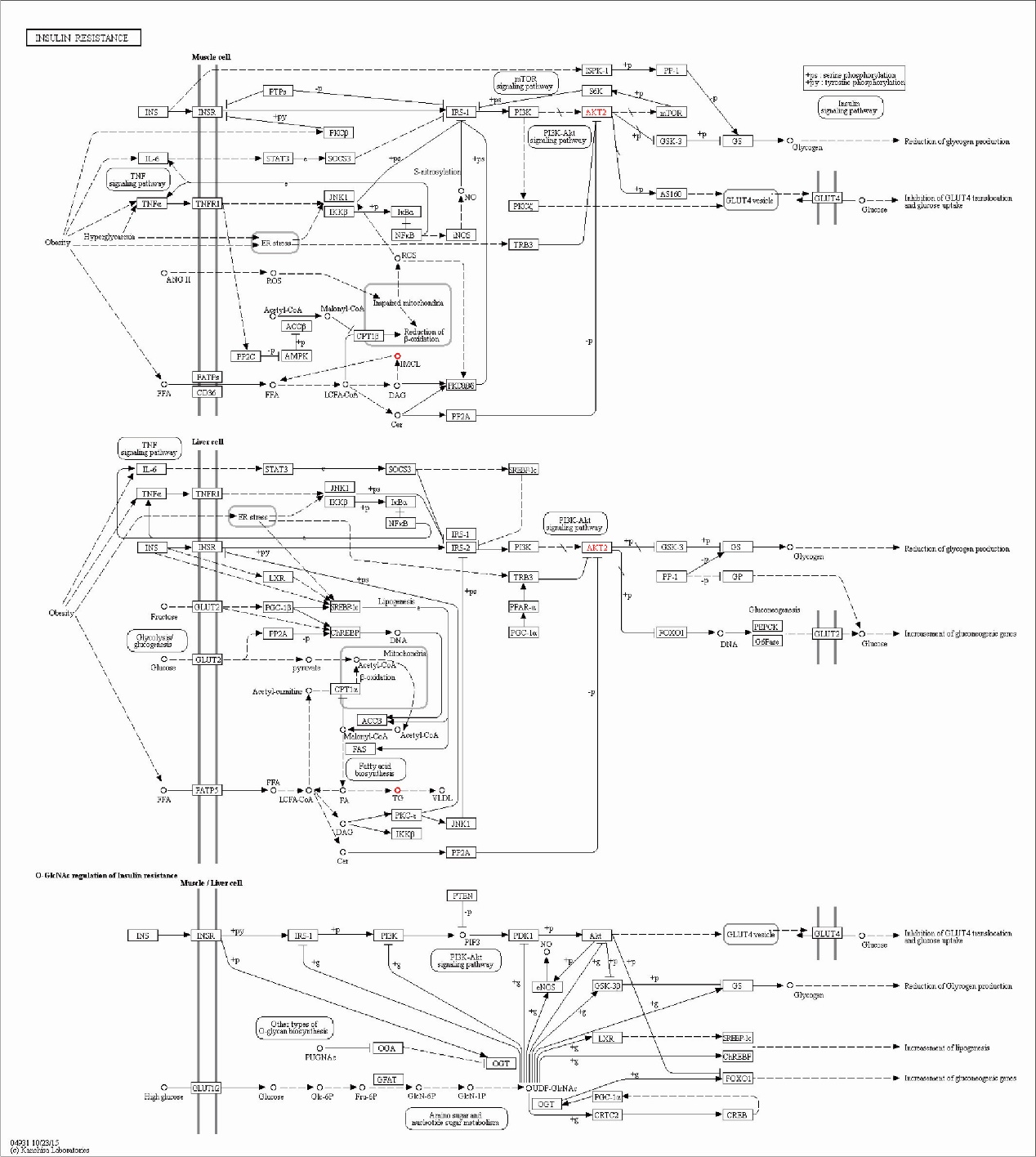


**Figure S4. Details of insulin resistance pathway.**


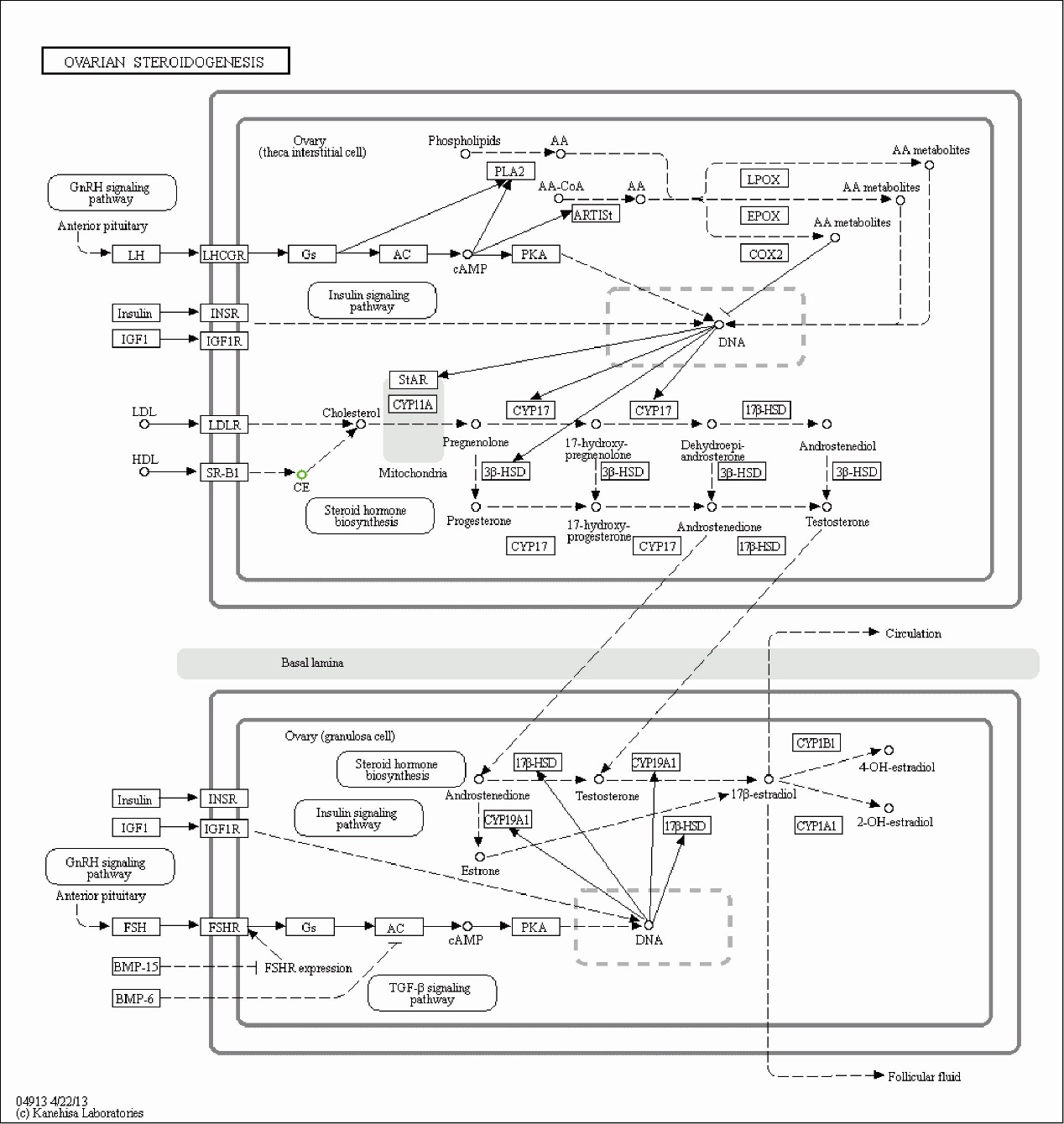


**Figure S5. Details of ovarian steroidogenesis pathway.**


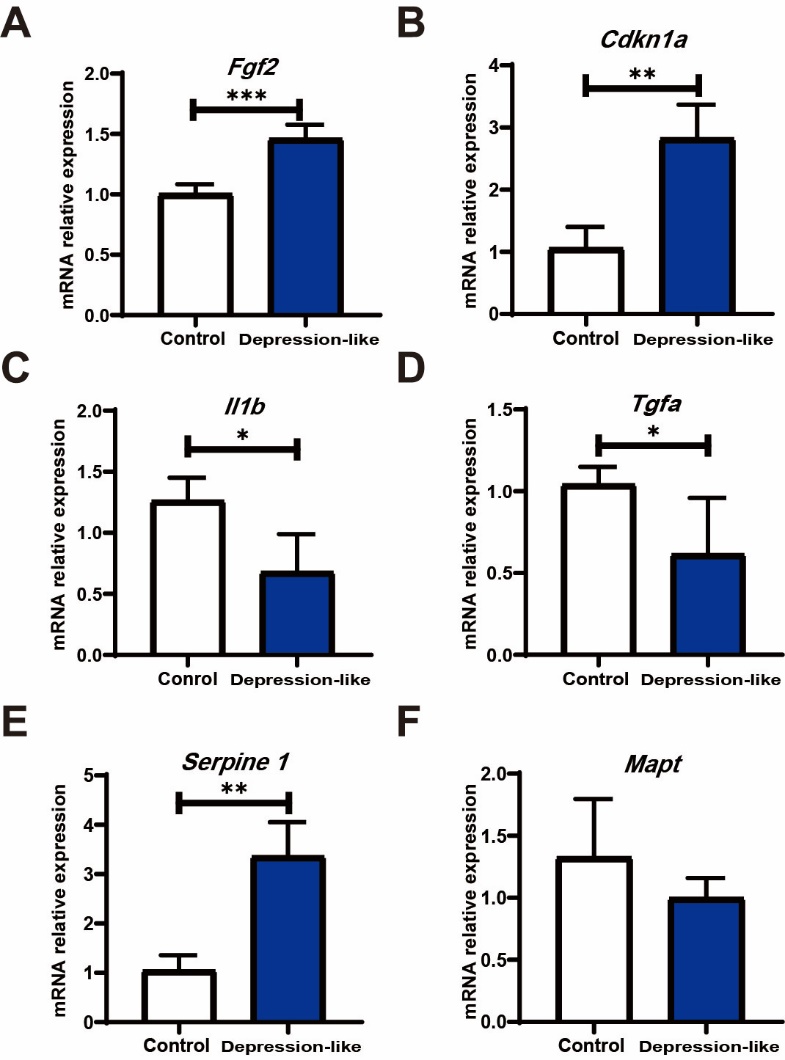


**Figure S6.** **The validation of differentially expressed mRNAs. A-F.** Expression levels of *Fgf2*, *Cdkn1a, Il1b*, *Tgfa*, *Serpine 1* and *Mapt*. The values are presented as the mean±SEM. Paired student t-test was adopted to analyze for significance. *P＜0.05, **P＜0.01.
